# Integrating Culture-based Antibiotic Resistance Profiles with Whole-genome Sequencing Data for 11,087 Clinical Isolates

**DOI:** 10.1016/j.gpb.2018.11.002

**Published:** 2019-05-14

**Authors:** Valentina Galata, Cédric C. Laczny, Christina Backes, Georg Hemmrich-Stanisak, Susanne Schmolke, Andre Franke, Eckart Meese, Mathias Herrmann, Lutz von Müller, Achim Plum, Rolf Müller, Cord Stähler, Andreas E. Posch, Andreas Keller

**Affiliations:** 1 Chair for Clinical Bioinformatics, Saarland University, 66123 Saarbrücken, Germany; 2 Institute of Clinical Molecular Biology, Christian-Albrechts University of Kiel, 24105 Kiel, Germany; 3 Siemens Healthcare GmbH, Strategy and Innovation, 91052 Erlangen, Germany; 4 Department of Human Genetics, Saarland University, 66421 Homburg, Germany; 5 Institute of Medical Microbiology and Hygiene, Saarland University, 66421 Homburg, Germany; 6 Ares Genetics GmbH, 1030 Vienna, Austria; 7 Curetis GmbH, 71088 Holzgerlingen, Germany; 8 Department of Pharmacy, Pharmaceutical Biotechnology, Saarland University, 66123 Saarbrücken, Germany; 9 Department of Microbial Natural Products, Helmholtz-Institute for Pharmaceutical Research Saarland (HIPS), Saarland University, 66123 Saarbrücken, Germany; 10 Helmholtz Center for Infection Research and Pharmaceutical Biotechnology (HZI), Saarland University, 66123 Saarbrücken, Germany

**Keywords:** Antibiotic resistance, Whole-genome sequencing, Bacteria, Pan-genome

## Abstract

Emerging **antibiotic resistance** is a major global health threat. The analysis of nucleic acid sequences linked to susceptibility phenotypes facilitates the study of genetic antibiotic resistance determinants to inform molecular diagnostics and drug development. We collected genetic data (11,087 newly-sequenced whole genomes) and culture-based resistance profiles (10,991 out of the 11,087 isolates comprehensively tested against 22 antibiotics in total) of clinical isolates including 18 main species spanning a time period of 30 years. Species and drug specific resistance patterns were observed including increased resistance rates for *Acinetobacter baumannii* to carbapenems and for *Escherichia coli* to fluoroquinolones. Species-level **pan-genomes** were constructed to reflect the genetic repertoire of the respective species, including conserved essential genes and known resistance factors. Integrating phenotypes and genotypes through species-level pan-genomes allowed to infer gene–drug resistance associations using statistical testing. The isolate collection and the analysis results have been integrated into GEAR-base, a resource available for academic research use free of charge at https://gear-base.com.

## Introduction

The development of new antimicrobial drugs has largely stagnated over the last few decades [1], while the drug resistance rates of many pathogens have at the same time been increasing [2], [3], [4]. Various large-scale efforts have been launched to investigate the emerging drug resistance, such as the Meropenem Yearly Susceptibility Test Information Collection (MYSTIC) program [2], the Canadian National Intensive Care Unit (CAN-ICU) study [5], the Canadian National Surveillance (CANWARD) study [6], [7], the Center for Disease Dynamics, Economics and Policy (CDDEP) study [3], and the European Antimicrobial Resistance Surveillance Network (EARS-Net) survey [8]. The results of these studies have shed light on the most common bacterial pathogens and resistance rates for regularly administered antibiotics, with the primary focus on the trend analysis of specific bacterial groups, periods of time, or locations [2], [3], [9], [10], [11], [12]. The global challenge of emerging drug resistance is further exacerbated by the rising prevalence of microorganisms with multidrug resistance (MDR) phenotypes [13]. Accordingly, identifying and administering the most effective drug in each individual case is of even greater importance for successful treatment of bacterial infections. However, these studies did not investigate the genetic repertoire of the pathogens, which represents an important source of information—*e.g.*, the resistance genotype may be readily revealed while the respective phenotype is misleading or not expressed under artificial laboratory conditions [14], [15].

Simultaneously, the recovery of genomic information from microorganisms via high-throughput sequencing approaches has become a routine task. This not only allows the high-resolution study of individual organisms’ genomes, but also the aggregated study in the form of “pan-genomes”—the united genetic repertoire of a clade [16]. Pan-genomes can be used to identify common genetic potential—*i.e.*, the “core” genes of a clade—as well as genes that are less broadly conserved (“accessory” or “singleton” genes) [16]. This facilitates the identification of essential genes or genes that provide adaptation advantages. Multiple computational approaches are available for the systematic creation of pan-genomes, *e.g.*, Roary [17], EDGAR [18], and panX [19]. As a result, a variety of bacterial pan-genomes, typically at the species-level, have thus far been constructed [20], [21], [22], [23]. However, most pan-genome studies focus on distinct species and do not always cover clinically relevant species. For example, MetaRef represents a resource that provides information about pan-genomes from multiple species and integrates approximately 2800 public genomes [24]. Although the diversity of the therein included organisms is particularly broad, the depth is limited in relation to clinically relevant bacteria—*e.g.*, seven *Klebsiella pneumoniae* genomes. Moreover, individual isolates included in the studies often span narrow time frames and/or have limited geographic spread.

While pan-genomic studies typically focus on the genetic information alone, efforts combining genomic and phenotypic information, in particular from antibiotic resistance testing, for the study of conserved or emerging resistance mechanisms are becoming increasingly prevalent [25], [26], [27], [28]. There are many antibiotic resistance resources available [29], however only few link genomic and phenotypic information of bacterial isolates. One of such resources is the Pathosystems Resource Integration Center (PATRIC) [30], which represents a rich service for the study of >80,000 genomes [31]. Yet, antimicrobial resistance information is available only for about 10% of the genomes. Furthermore, as the genomes and the associated metadata of PATRIC are imported from public resources, which are populated by individual research efforts, data standardization or normalization is challenging. Finally, individual taxa may be underrepresented and thus warrant expansion—*e.g.*, the number of *Escherichia spp.* genomes with antimicrobial resistance metadata is almost two orders of magnitude smaller than that of *Mycobacterium spp.* genomes [31].

**Figure 1:**
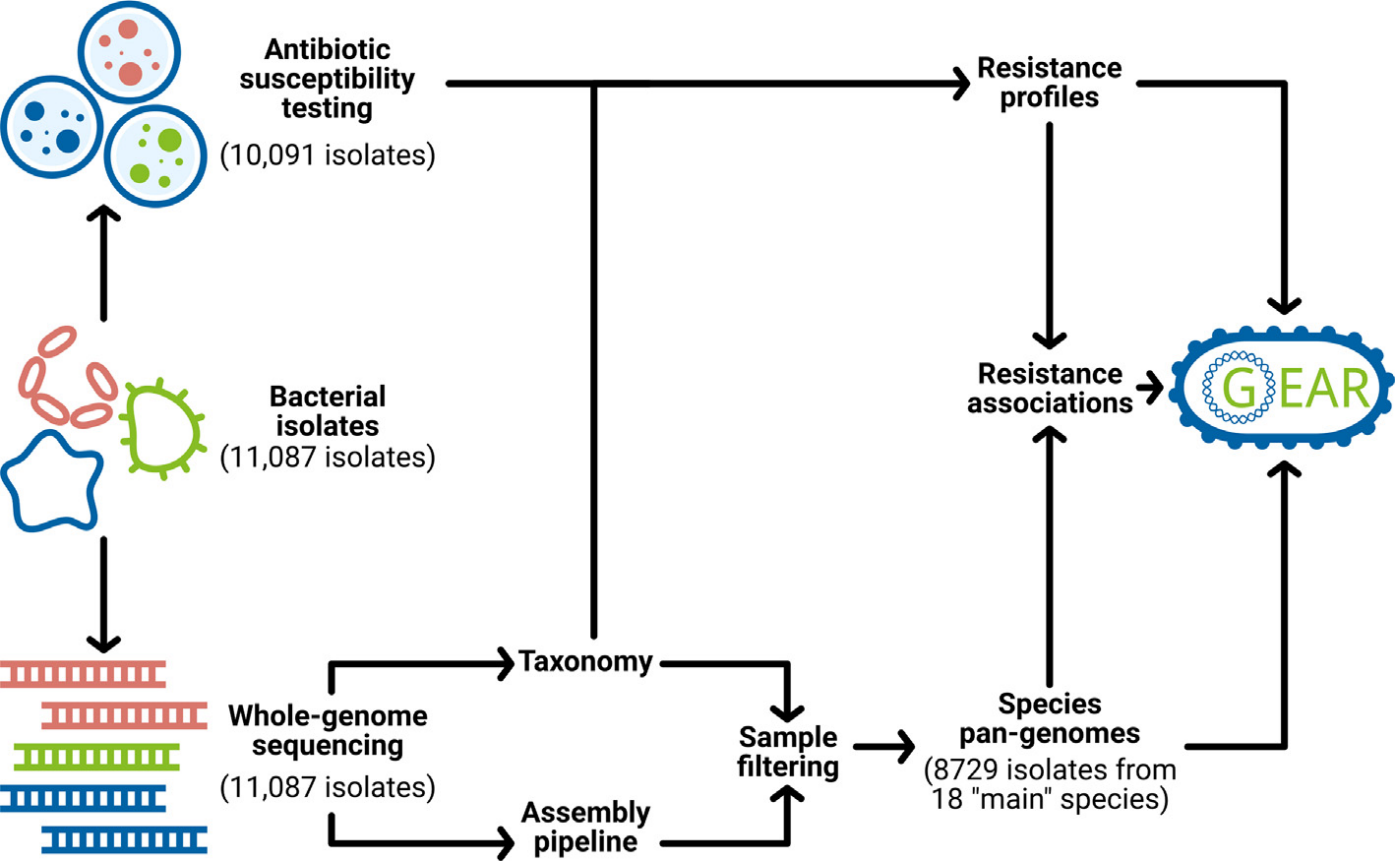
**GEAR-base workflow and structure** Schematic overview of data collection, processing and integration into GEAR-base.

Motivated by the importance of linking resistance phenotypes with genomic features, we collected whole-genome sequencing data of 11,087 clinical isolates representing, *inter alia*, 18 main bacterial species. The samples were collected in North America, Europe, Japan, and Australia over a period of 30 years, and processed in a concerted effort, thereby reducing experimental bias. Culture-based resistance testing was performed for 10,991 out of the 11,087 isolates against 22 antibiotic drugs. Furthermore, species-level pan-genomes were constructed on the basis of per-isolate *de novo* assemblies and were used to infer gene–drug resistance associations. This wealth of information is integrated into an online resource, Genetic Antibiotic Resistance resource, or in short, GEAR-base (Figure 1). Providing broad organismal, antibiotic treatment and temporal coverage, GEAR-base is expected to support the pan-genome-based study of bacteria and to advance research on known or emerging antibiotic resistance mechanisms. GEAR-base is available for academic research use free of charge at https://gear-base.com.

## Results

### Resistance testing of cultured bacterial isolates

**Figure 2:**
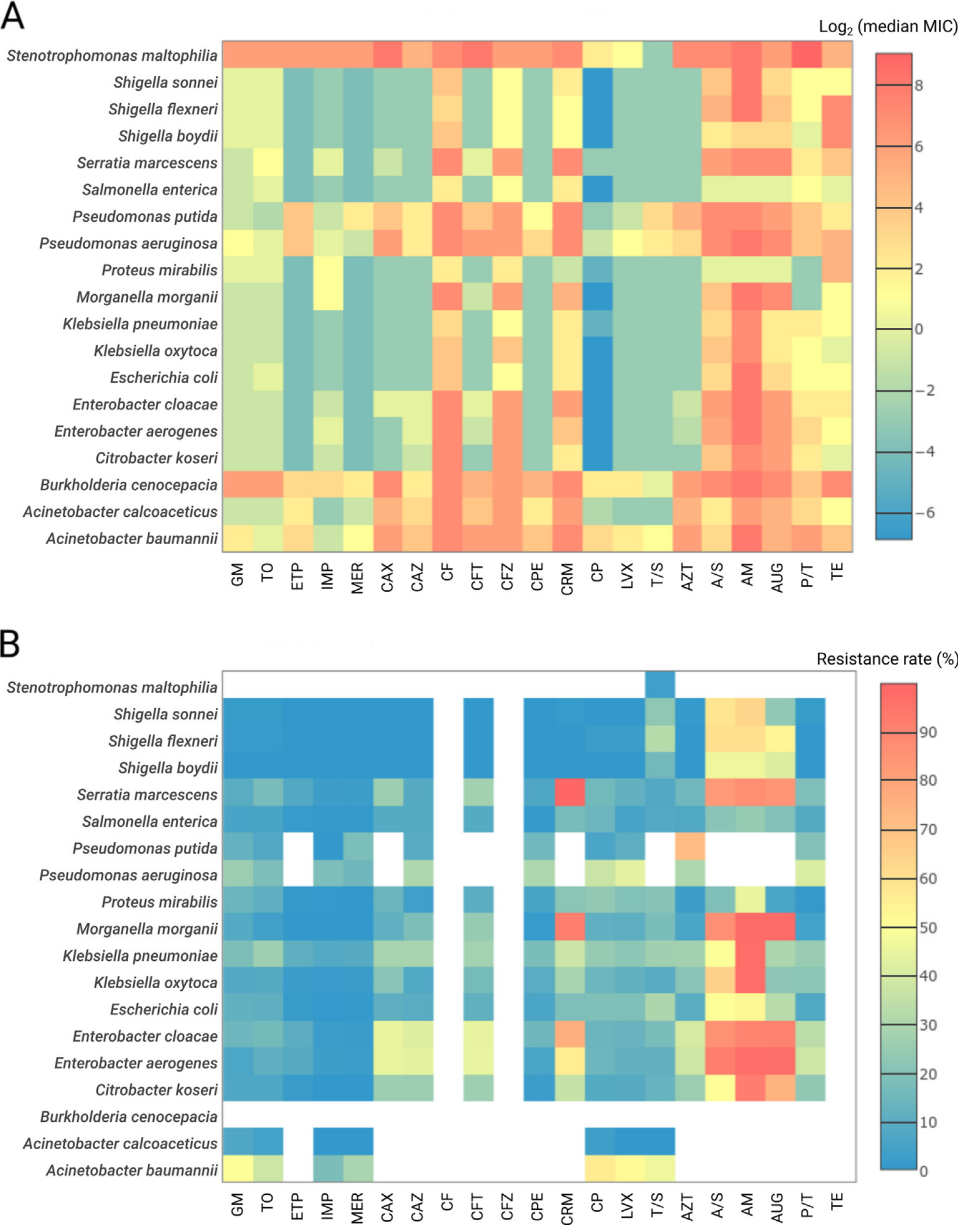
**Overview of resistance profiles** Heatmaps of log-transformed (base 2) median MIC values (**A**) and resistance rates (**B**) for all species with at least 50 isolates. Drugs labels were grouped relative to their class. The cells are coded in color gradient from blue to red with blue for lower values and red for higher values. White color in panel B corresponds to the cases where no breakpoints are available from the used guidelines. MIC, minimum inhibitory concentration.

The present dataset of 11,087 bacterial isolates covered a total of 6 families, 14 genera, and 20 species (considering species with at least 50 isolates, Table S1) and comprised two datasets: 1001 isolates from the *Staphylococcus aureus* strain collection and 10,086 isolates from the Gram-negative collection. From the *S*. *aureus* strain collection, 993 isolates were tested for methicillin resistance and susceptibility (see Methods section). For 9998 isolates from the Gram-negative collection, culture-based antimicrobial susceptibility testing (AST) for 21 commonly-prescribed Food and Drug Administration (FDA)-approved antibiotics from 8 drug classes was performed to determine the respective minimum inhibitory concentrations (MICs) (Figure 2A). The resistance profiles were determined for each isolate in accordance with the European Committee on Antimicrobial Susceptibility Testing (EUCAST) guidelines (v. 4.0) for a total of 182 drug concentrations (7–11 concentrations per drug; Tables S2 and S3, Figure 2B). Whole-genome sequencing (WGS)-based taxonomic identification was performed for all isolates [32]. In the following content, we focused on the analysis results of the MICs and resistance profiles of the 9998 isolates from the Gram-negative collection.

**Supplementary Figure S1:**
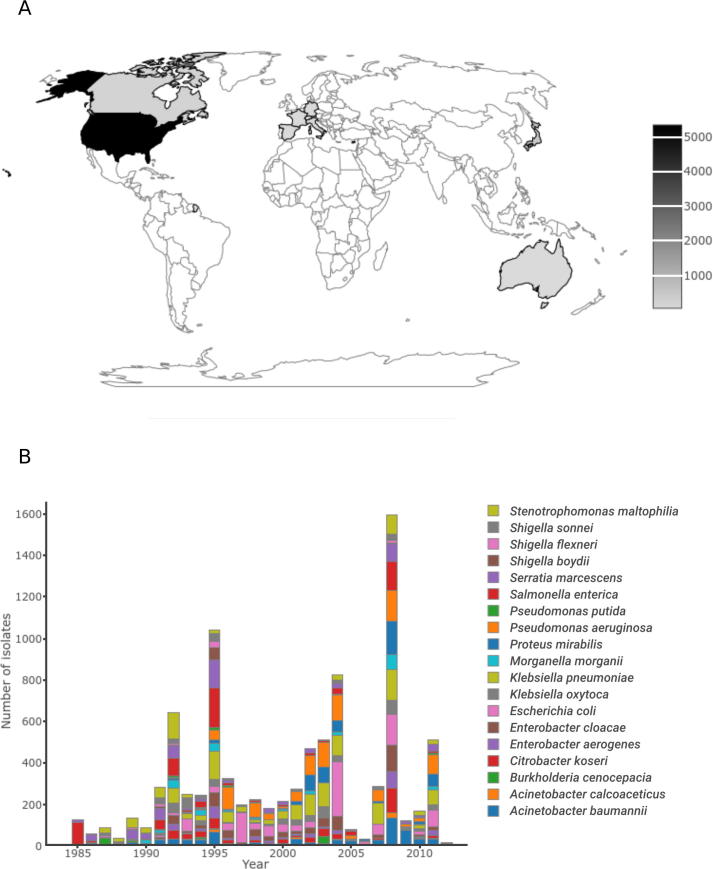
**Local and temporal distribution of the bacterial isolates** The numbers of bacterial isolates collected in different countries (A) and years (B) for species with at least 50 isolates.

All patient-derived isolates were collected in clinics located in North America, Europe, Japan, or Australia from 1983 to 2013 (Figure S1). Varying degrees of resistance were observed among the isolates (Figure 2B). The majority of species demonstrated relatively low resistance rates (<20%) to aminoglycosides (gentamicin and tobramycin) and carbapenems (ertapenem, imipenem, and meropenem), except for *Acinetobacter baumannii* (≥ 29% for aminoglycosides and meropenem), *Pseudomonas aeruginosa* (26% for gentamicin), and *Klebsiella pneumoniae* (26% for tobramycin). These rates were compared against two independent large-scale studies—CDDEP (USA-based results; CDDEP ResistanceMap, https://resistancemap.cddep.org/AntibioticResistance.php, accessed on September 26, 2017) [3] program and the MYSTIC program [2], for matching species and drug data. Both studies report low (<20%) resistance rates for the aminoglycosides and carbapenems during the observation period (1999–2012/2014 for CDDEP and 1999–2007/2008 for MYSTIC) except for *A*. *baumannii* (CDDEP: >20% since 2005 for carbapenems and >35% during 1999–2012 for aminoglycosides; MYSTIC: >37% in 2007/2008 for carbapenems and >20% during most years for aminoglycosides). For *K*. *pneumoniae* and tobramycin (aminoglycosides for CDDEP), MYSTIC and CDDEP reported >10% resistance rates since 2005 with only one value of above 20% observed by MYSTIC in 2007. Finally, for *P*. *aeruginosa* and gentamicin, MYSTIC reported a resistance rate of only around 10%. The rate of isolates resistant to multiple antibiotic drugs, *i.e.*, resistant to at least three drugs from different drug classes (CDDEP ResistanceMap), was highest for *A. baumannii* (44%) and for *Enterobacter* spp. (41%–45%). For the remaining species and drug classes, the MDR rates were at least 20%, except for *Acinetobacter calcoaceticus* (0%), *Salmonella enterica* (11%), and *Shigella* spp. (0%–3%). In addition to the investigation of individual species–drug combinations, we analyzed whether drug pairs showed correlating MIC profiles among all isolates (Figure S2). In general, the highest correlations were expectedly found within separate drug classes—*e.g.*, for fluoroquinolones, aminoglycosides, and carbapenems. While for some species, *e.g.*, *Burkholderia cenocepacia*, a clear clustering according to drug classes and their mechanism of action was observed, other species, such as *S*. *enterica*, showed less pronounced cluster structures.

**Supplementary Figure S3:**
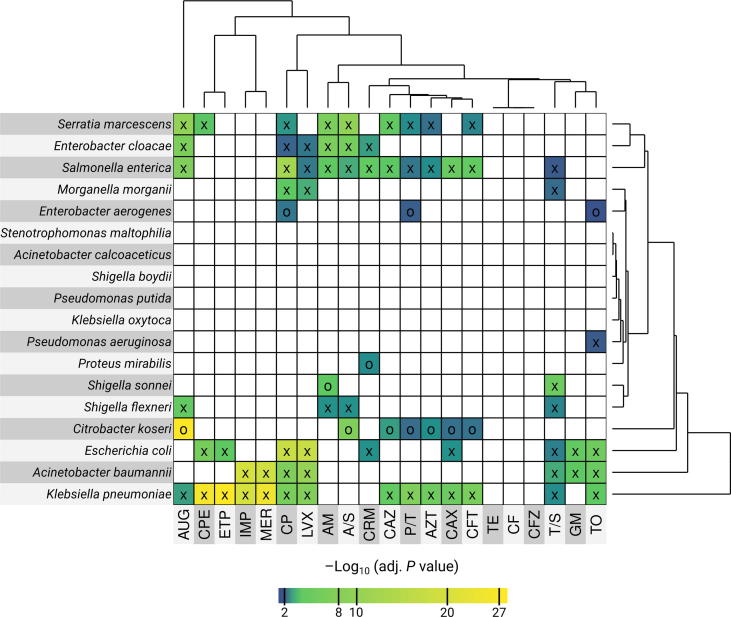
**Overview of results of WMW test comparing temporal distribution of non-resistant and resistant isolates** Two-sided WMW test comparing collection year distributions between non-resistant and resistant isolates. Only isolates with collection dates available from species with at least 50 isolates were included. Heatmap is coded in color gradient to indicate the negative log-transformed (base 10) FDR-adjusted P values from blue to yellow. The taxa and drugs were grouped by hierarchical clustering (Euclidean distance and average linkage on transformed P values). White cells indicate non-significant adjusted *P* values (≥0.01) or omitted tests (e.g., due to insufficient group size). Cases where the test estimate was below zero were marked with “x”, otherwise with “o”. FDR, false detection rate.

**Supplementary Figure S4:**
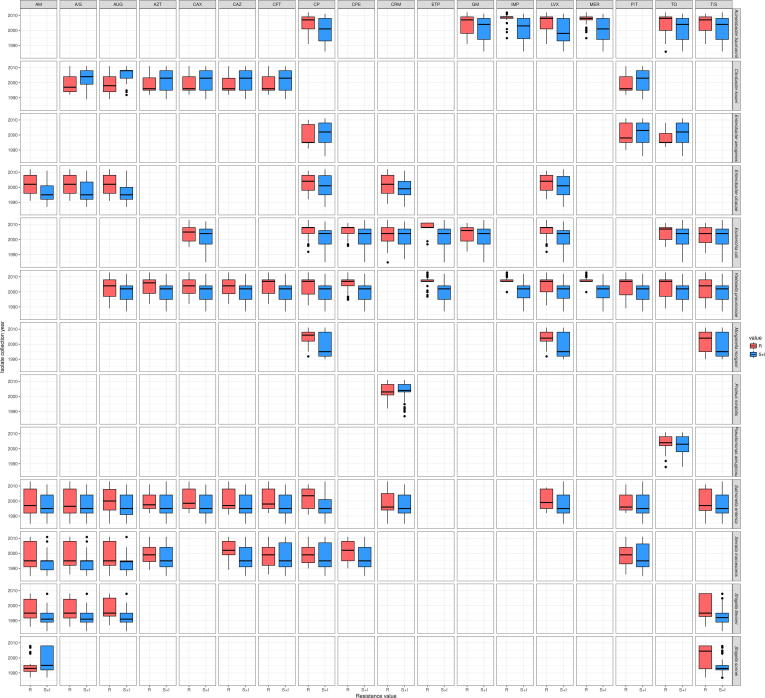
**Boxplots of the temporal distribution of non-resistant and resistant isolates** Boxplots of collection year for the non-resistant (blue) and resistant (red) isolates for all species–drug combinations with a significant result (adjusted *P* < 0.01). Only isolates and drugs used for comparison using the two-sided WMW test are shown.

Subsequently, we compared resistant and non-resistant isolates with respect to their collection year in order to identify potential trends of de-/increasing antibiotic resistance rates (Figures S3 and S4, and Table S4). The following species–drug pairs were found to exhibit particularly low *P* values [WMW-test, false discovery rate (FDR) adjusted *P* < 1E−17], as well as increases in resistance over time: *K*. *pneumoniae* to cefepime, *K*. *pneumoniae* and *A*. *baumannii* to carbapenems, and *E. coli* to fluoroquinolones. Similar trends were reported by the CDDEP [3] program (CDDEP ResistanceMap) and the MYSTIC program [2], including increasing resistance rates for *A*. *baumannii* to carbapenems (43% from 1999 to 2014 in the USA, CDEEP), and for *E*. *coli* to fluoroquinolones (30% from 1999 to 2014 in the USA, CDEEP; >20% from 1999 to 2008, MYSTIC).

While the culture-based analyses provide species-resolved information about resistance rates over time and corroborate previous findings on the global increase in antibiotic resistance, genetic features represent important factors and were thus concomitantly considered.

### Whole-genome *de novo* assembly of isolates and species pan-genomes

**Figure 3:**
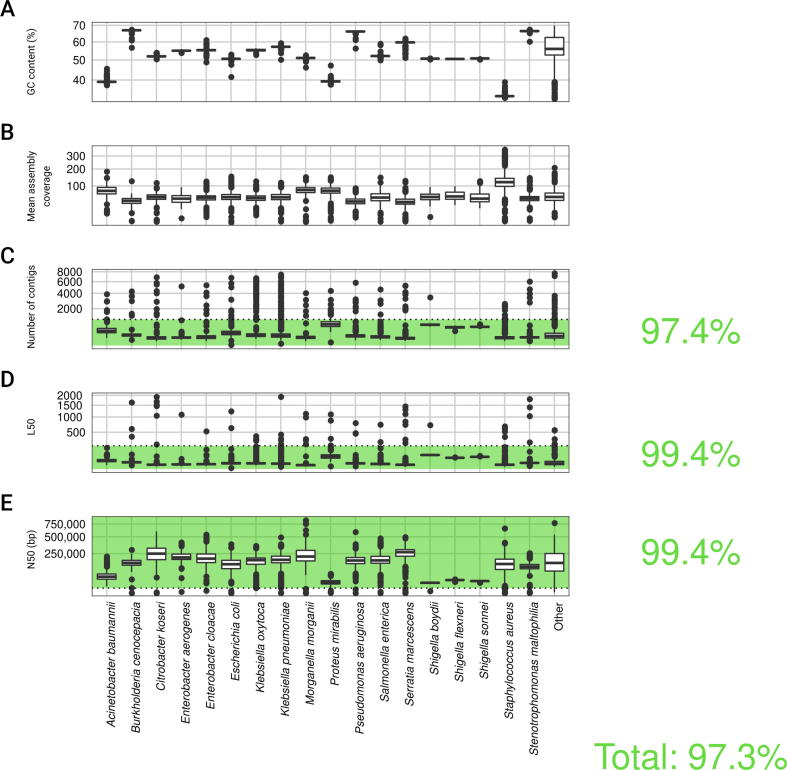
**Assembly quality overview** Assembly summary statistics for the 11,062 isolates with a *de novo* assembly. The isolates were grouped by their species taxon, and isolates not belonging to any of the main 18 species used for pan-genome construction were grouped into ”Other”. The box plots show the GC content (**A**), mean assembly coverage (**B**), number of contigs (**C**), L50 value (**D**), and N50 value (**E**) for contigs of at least 200 bp. The assembly quality cut off values are illustrated by dotted lines (1000 for the number of contigs; 200 for L50; and 5000 bp for N50). The plot area satisfying the respective filtering criterion is colored in green. Percentages of isolates passing the respective criterion as well as all criteria are shown to the right.

A total of 11,087 bacterial isolates were whole-genome sequenced using Illumina Hiseq2000/2500 sequencers, resulting in a median number of 1,517,147 paired reads per isolate (1,609,533 ± 620,481). *De novo* assemblies were successfully created for 11,062 (99.8%) isolates (Figure 3) and of these, the assembled genomes of 10,764 (97.3%) isolates passed the stringent assembly quality criteria. Moreover, the assembled genomes of 9206 (83% of 11,087) isolates fulfilled the quality criteria for taxonomic assignment. A total of 8729 isolates, representing 18 main species having ≥50 isolates, were used after stringent quality filtering (see Methods for sample filtering details) in the subsequent analyses and in the construction of species-level pan-genomes (Table S3).

**Supplementary Figure S5:**
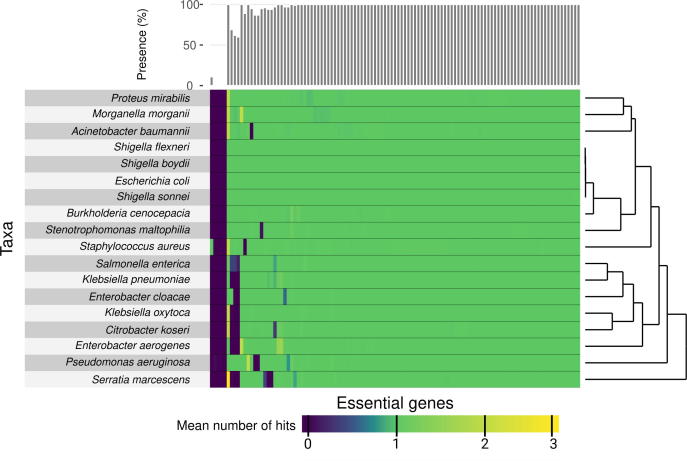
**Frequency of essential genes per species** Shown is the mean number of HMM hits coded in color gradient from blue to yellow. Rows (species) and columns (essential genes) were ordered using hierarchical clustering (Euclidean distance and average linkage). The bar plot at the top of the heatmap shows the frequency of the essential genes over all isolates. HMM, hidden Markov model.

**Supplementary Figure S6:**
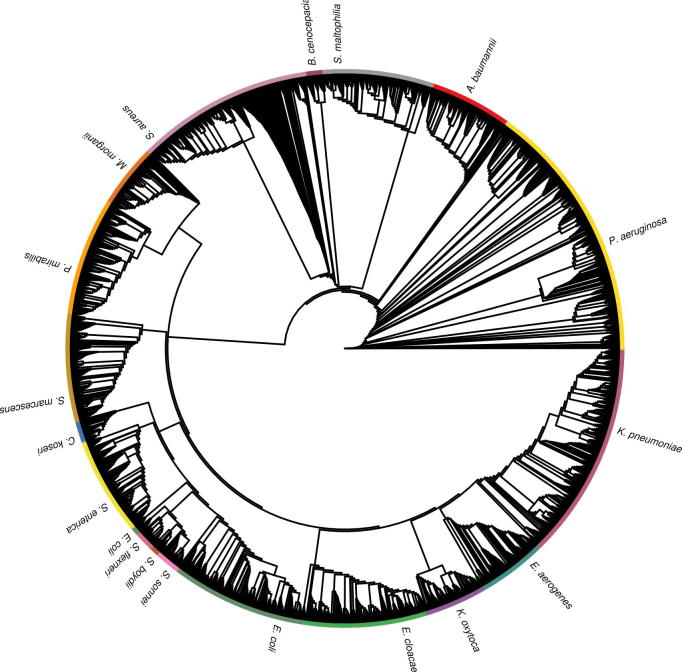
**Phylogenetic tree of the isolates** The phylogenetic tree of the isolates built using RAxML and multiple sequence alignments of the best hits of the essential genes found in at least 99% of the isolates. The tree tips are colored relative to the isolate species taxon.

**Supplementary Figure S7:**
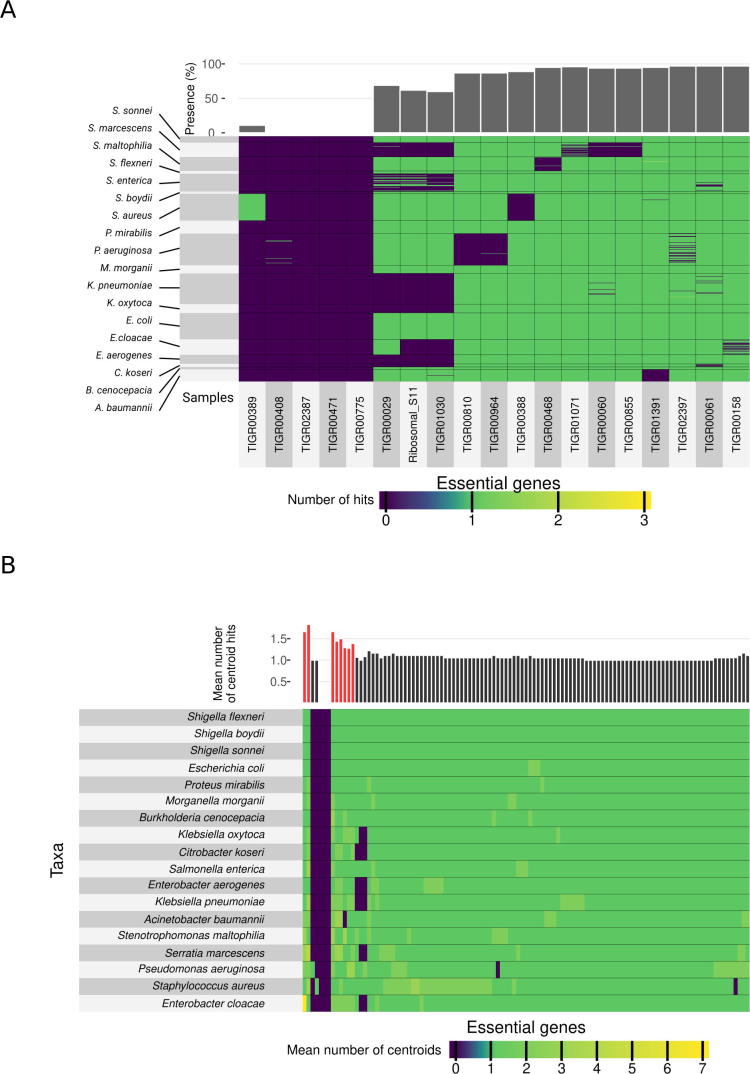
**Essential gene hits per isolate and number of centroids covering essential genes A.** Essential gene hits per isolate found in less than 99% of the isolates. The number of hits per isolate is coded in color gradient and the isolates were grouped by species. The bar plot at the top of the heatmap shows the frequency of the essential genes over all isolates. **B.** Mean number of centroids containing genes matched to essential genes per species. The bar plot shows the mean across all species and bars representing the mean number larger than 1.25 are highlighted in red.

First, the presence/frequency of genes from a set of 111 single-copy marker genes, which were defined as essential genes by Dupont et al. [33], was used as a proxy to estimate the genome completeness of individual *de novo* assemblies. Overall, the assemblies were found to be largely complete. 92 essential genes (82.9%) were identified in at least 99% of the 8729 isolates (Figure S5) that were used to construct a phylogenetic tree of these isolates (Figure S6). Furthermore, species-specific presence/absence patterns were frequently observed (Figure S7A). For example, TIGR00389 (glycine–tRNA ligase) was only found in *S. aureus*, whereas TIGR00388 (glycine–tRNA ligase, alpha subunit) was not present in this species. Four genes, TIGR00408 (encoding the proline–tRNA ligase), TIGR02387 (encoding the DNA-directed RNA polymerase, gamma subunit), TIGR00471 (encoding the phenylalanine–tRNA ligase, beta subunit), and TIGR00775 (encoding the Na^+^/H^+^ antiporter, NhaD family), were not found in any of the isolates, except for sporadic hits in *Pseudomonas aeruginosa* for TIGR00408.

**Supplementary Figure S8:**
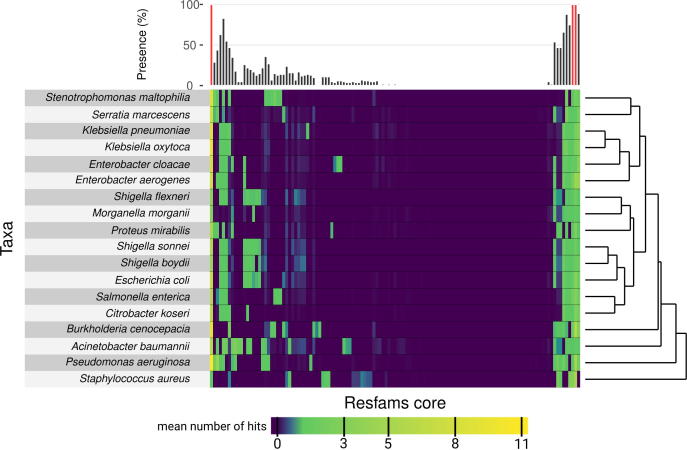
**Frequency of Resfams hits per species** Shown is the mean number of hits per resistance factor from the Resfams core database. The rows and columns were ordered using hierarchical clustering (Euclidean distance and average linkage). The bar plot at the top of the heatmap shows the frequency of the resistance factor across all isolates and the bars showing frequencies of at least 90% are highlighted in red.

In the next step, Resfams core-based resistance factors [34] were annotated in the isolate assemblies in order to study the species-level distribution of these genetic features. The number of covered Resfams (mean count of hits ≥1) varied between species from 4.1% (5 of 123 Resfams, *Morganella morganii*) to 11.4% (14 of 123 Resfams, *A*. *baumannii* and *Shigella sonnei*) (Figure S8). Three Resfams were found in at least 90% of all considered isolates. These are all antibiotic efflux pumps, which include RF0007 [ATP-binding cassette (ABC) type], RF0107 (ABC type), and RF0115 [resistance-nodulation-cell division (RND) type], with the latter having a mean count of hits of ≥5 for 14 out of 18 species.

The multi-locus sequence typing (MLST) analysis revealed, that in all species with a typing scheme included in the used version of PubMLST, isolates were assigned to at least 6 different sequence types (STs), except for *S*. *sonnei*, and new STs could be identified, except for *Shigella flexneri* and *S*. *sonnei* (Figure S9). Among these species, the proportion of isolates without a confident assignment was high (≥10%) for *B*. *cenocepacia*, *Enterobacter cloacae*, *Klebsiella oxytoca*, and *Stenotrophomonas maltophilia*.

**Figure 4:**
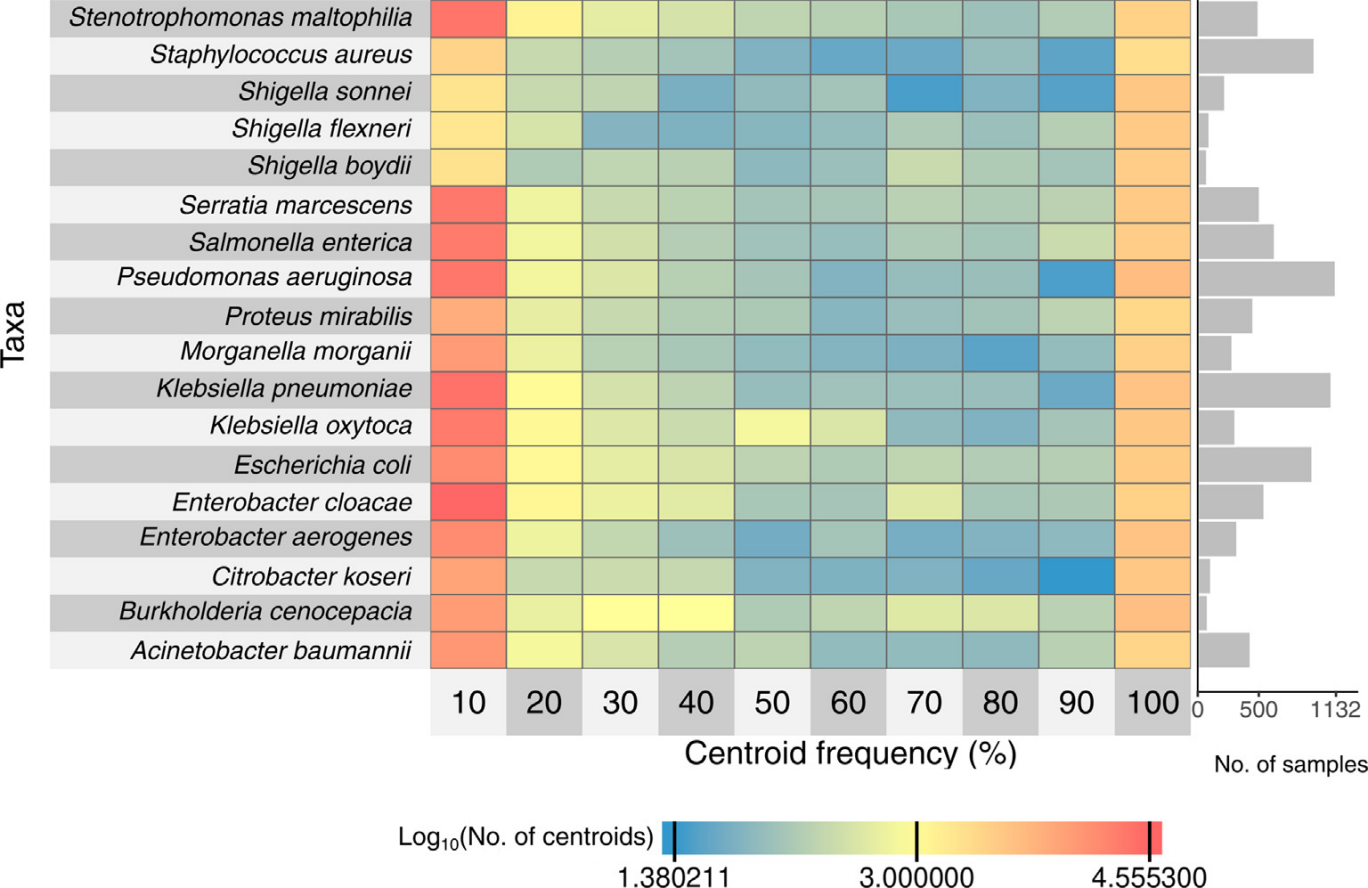
**Centroid frequency** Number of centroids in each pan-genome of the 18 main species in relation to their frequency. The first column contains centroids that are present in <10% of the isolates, and the last one contains centroids that are present in ≥90% of the isolates. Cells are coded in color gradient to indicate the log10-transformed number of centroids. The bar plot on the right shows the number of isolates used to construct the respective pan-genomes.

**Supplementary Figure S10:**
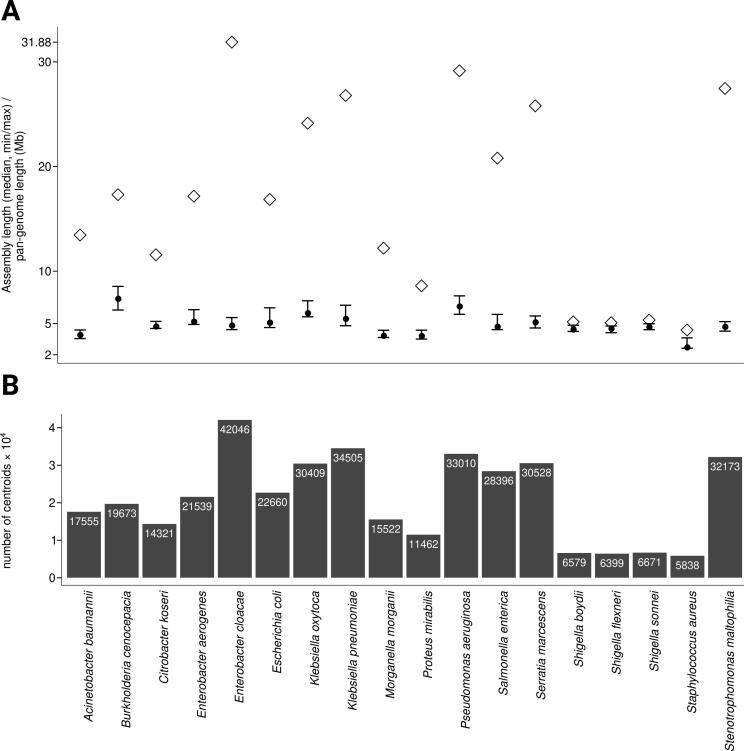
**Assembly length and pan-genome size A.** Median assembly length (solid black points), minimal and maximal assembly length (error bars), and pan-genome size (cumulative length over all centroids, diamond-shaped points) in Mb. **B.** Number of centroids per pan-genome.

**Supplementary Figure S11:**
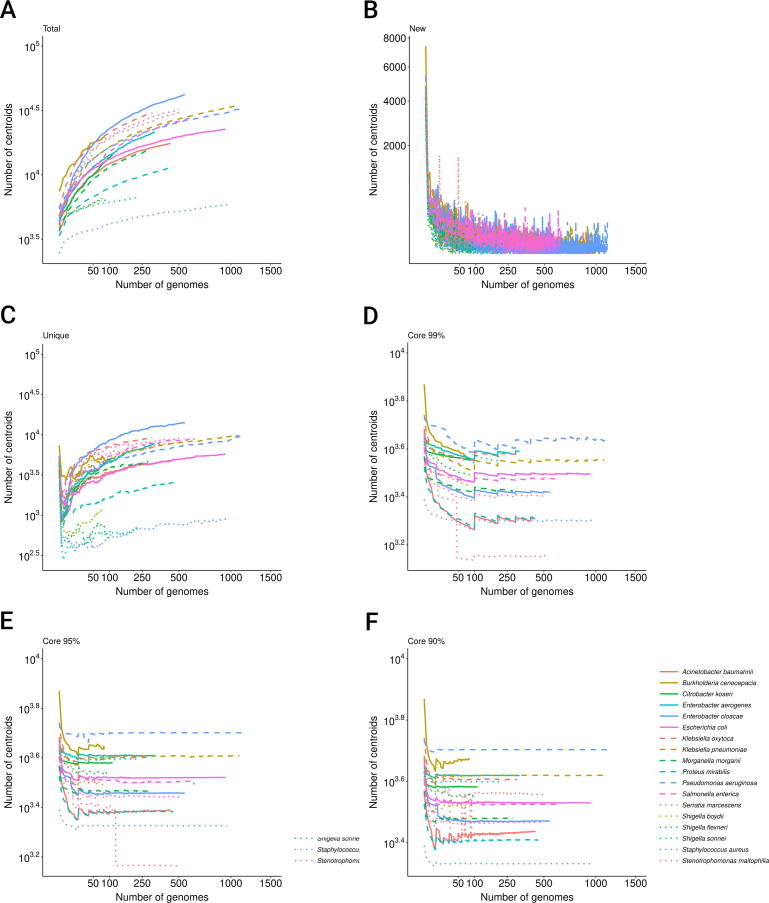
**Estimated centroid rates for all pan-genomes** The plots show the centroid rates of all (**A**), unique (**B**), new (**C**), and core (99%, 95%, and 90%) centroids (**D–F**) as a function of the number of genomes (isolates) included in the pan-genome. The rates were estimated from 100 permutations of the centroid presence/absence matrices as the median value over all permutations performed.

**Supplementary Figure S12:**
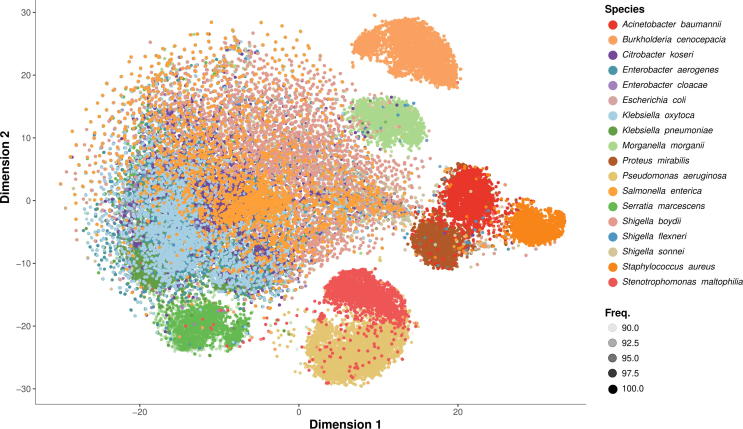
**Two-dimensional embedding of pan-genome centroids per species** Centroids with a isolate frequency ≥90% are shown. The respective species of the centroids’ source pan-genome were color coded.

The size of the species pan-genomes (*i.e.*, the number of centroids) ranged from 5838 (*S*. *aureus*, total pan-genome length <5 Mb) to 42,046 (*E. cloacae*, total pan-genome length >30 Mb) (Figure S10). A centroid refers here to the representative gene of a homologous gene cluster with ≥90% pair-wise amino acid sequence identity (Methods). Most centroids were found in <10% or in ≥90% of the isolates (Figure 4). Moreover, all pan-genomes were found to be open based on the analysis of the number of centroids in relation to the number of included genomes (Figure S11, Table S5). The two-dimensional embedding of the core centroids from the pan-genomes revealed many taxon-specific patterns (Figure S12) with distinct clusters for *B*. *cenocepacia*, *M*. *morganii*, *A*. *baumannii*, *Proteus mirabilis*, *S*. *aureus*, *S*. *maltophilia*, *P*. *aeruginosa*, and *Serratia marcescens*. We compared the number of (core) centroids in our pan-genomes to the numbers reported by panX [19] (http://pangenome.tuebingen.mpg.de, accessed on January 29, 2018). The number of centroids present in at least 90% of the analyzed genomes was consistent for all matching species (Table S6). However, the pan-genome size, *i.e.*, the total number of centroids described in GEAR-Base, was similar for *E*. *coli* and *S*. *aureus*, but exceeded substantially the number of centroids described in panX for *A*. *baumannii*, *K*. *pneumoniae*, *P*. *aeruginosa*, and *S*. *enterica* (Table S6). With respect to the presence of essential genes in the species-level pan-genomes, the mean number of centroids containing at least one matching gene was one, that is, these essential genes were mostly found in only one centroid cluster (Figure S7B). However, the mean number of centroids was ≥1.25 for eight essential genes, *i.e.*, in some species these genes were found in multiple centroid clusters.

In the following section, the resistance phenotypes and genomic features were linked and significantly associated centroids were further studied, with respect to their overlap to known resistance genes from the Resfams core database.

### Resistance associations by linking phenotype and genotype

**Figure 5:**
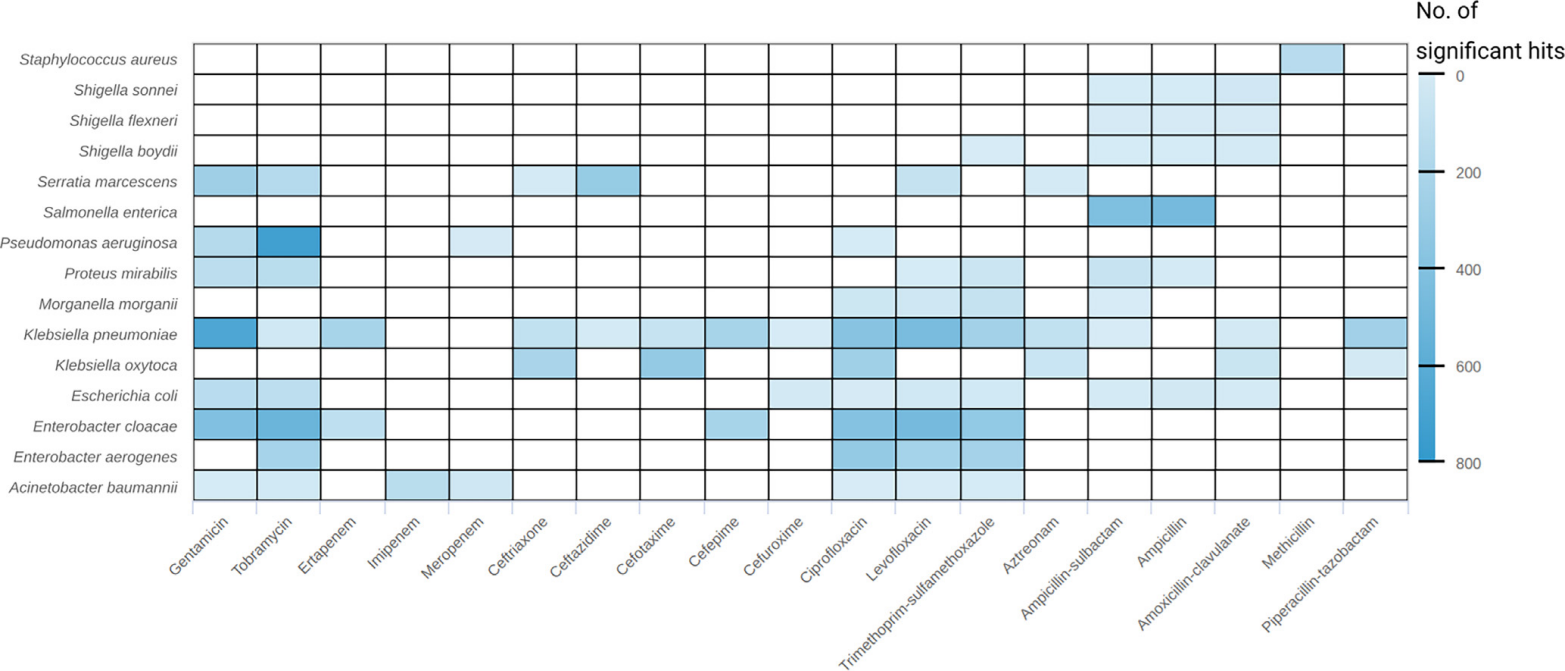
**Number of significant results of the resistance association analysis** Significant results (adjusted *P* < 1E−5) of the resistance association analysis based on the presence/absence of centroids. The heatmap shows the number of significant results (in color gradient with lighter blue for smaller numbers and darker blue for larger numbers) per taxon and drug. Drugs are sorted according to their class.

**Supplementary Figure S13:**
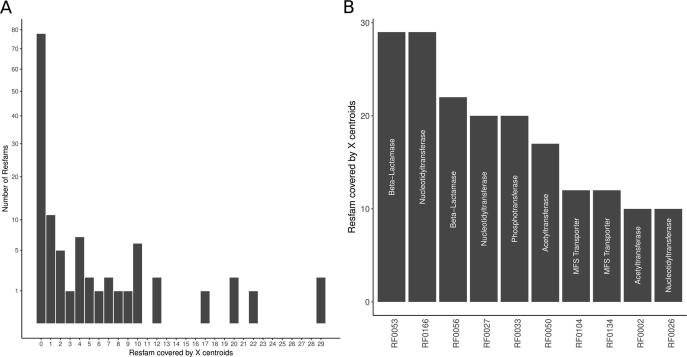
**Resfams covered by centroids associated with resistance** Number of centroids covering Resfams considering only centroids with a significant resistance association. **A.** Histogram of the centroid counts; the values on Y axis is square root transformed. **B.** The top 10 Resfams with their mechanism class and the number of centroids covering them.

We used binary information in the form of centroid presence/absence to test for significant centroid–drug associations per species. The number of found associations ranged from below 10 to above 500; most associations (≥500) were found for *P*. *aeruginosa* and tobramycin, and *K*. *pneumoniae* and gentamicin (Figure 5). Furthermore, the drug resistance-associated centroids encoding for a resistance gene were investigated. From the Resfams core database, 45 of the 123 factors were found in at least one centroid (Figure S13). Among these, the top ten Resfams genes from both analyses covered various resistance mechanism classes – nucleotidyltransferases, phosphotransferases, acetyltransferases, beta-lactamases, and major facilitator superfamily (MFS) transporters (Figure S13B).

### GEAR-base online resource

The GEAR-base resource is freely accessible at https://gear-base.com for academic research use and currently provides two modules for browsing of the database—a culture-based module and a pan-genome module—as well as a module for the analysis of user-provided data. The culture-based module is focused on the Gram-negative isolate collection and provides an interactive view of the taxonomic composition, MIC, and resistance profiles, as well as additional meta-data, *e.g.*, collection year or isolate distributions. The pan-genome module provides general statistics, such as assembly quality of the included isolates, pan-genome size, and resistance association analysis overview, for both the Gram-negative and the *S. aureus* isolates. Gene nucleotide sequences can be downloaded for each individual pan-genome centroid and a batch-download of all centroid nucleotide sequences is available. Moreover, pan-genome centroids can be browsed online for specific gene products and filtered by their presence in the isolates. In addition, centroid clusters can be viewed including associated gene annotations, the hits to the Resfams core database, and information about potential resistance associations against the set of herein included drugs. GEAR-base’s analysis module allows the user to query individual gene sequences against the pan-genome centroid sequences using Sourmash [35], against hidden Markov models (HMMs) of pan-genome centroid clusters and Resfams core database using HMMER (http://hmmer.org/), and against the NCBI nt/nr database using BLASTp [36]. Furthermore, a genome-scale search against the present clinical isolate collection, the finished genomes from the NCBI RefSeq database [37], as well as the National Collection of Type Cultures (NCTC) 3000 genomes project from the Public Health England and the Wellcome Trust Sanger Institute (http://www.sanger.ac.uk/resources/downloads/bacteria/nctc/, accessed on October 18, 2017) can be performed online using Mash/MinHash [38].

We used a recently-published *K*. *pneumoniae* genome [39] (strain 1756, NCBI assembly accession ID GCF_001952835.1_ASM195283v1) to demonstrate the analysis functionalities of GEAR-base. In a first step, the chromosome and plasmid sequences were uploaded and a perfect match was found to the genome’s NCBI entry, as expected. The next-best matches were to a *K*. *pneumoniae* isolate from the current collection of clinical isolates (828/1000 shared hashes, distance of 4.71E−3), and to a *Klebsiella* sp. genome (ERS706555) from the NCTC 3000 database (709/1000 shared hashes, distance of 8.89 E-3). In a second step, all coding DNA sequences (CDS) were searched against the pan-genome centroids in GEAR-base using Sourmash and against the Resfams core database. The majority of the pan-genome hits were related to *K*. *pneumoniae* (6206 hits of 11,267) followed by *E*. *aerogenes* (1537 hits) and *K*. *oxytoca* (1014 hits). *S. aureus*, a Gram-positive species, served as an outgroup and no hits to its pan-genome were found. In total, 37 hits to 21 unique Resfams (core database) were found in the query genome CDS with 23 hits on the chromosome and 14 on the plasmid. The top three most occurring Resfams were RF0115 (8 hits, RND antibiotic efflux pump), RF0098 (3 hits, multidrug efflux RND membrane fusion protein MexE, RND antibiotic efflux), and RF0053 (3 hits, class A beta-lactamase). Furthermore, the CDSs of eight antibiotic resistance genes reported in the original genome announcement were investigated. The HMM-based search of pan-genome centroids resulted in the identification of two chromosomal CDSs, WP_076027158.1 (multidrug efflux RND transporter periplasmic adaptor subunit OqxA) and WP_004146118.1 (FosA family fosfomycin resistance glutathione transferase), being classified as *K*. *pneumoniae*-derived centroids according to their top hits (with respect to the full sequence score). The top hits of the remaining genes (5 plasmid-derived and 1 chromosome-derived) included centroids from other Gram-negative species. However, the centroid cluster annotations matched the expected protein functions for all eight CDSs independent of the species. The top three hits for WP_004146118.1 were centroids from *K*. *pneumoniae*, *E*. *aerogenes*, and *K*. *oxytoca*, matching the expected annotation and present in almost all isolates (>98%) of the respective pan-genomes. This high prevalence matches the observations made by Ryota *et al*. reporting similarly high frequency (>96%) of *fosA* in these species [40]. For the beta-lactamases WP_004176269.1 (class A broad-spectrum beta-lactamase SHV-11) and WP_000027057.1 (class A broad-spectrum beta-lactamase TEM-1), the top hits in *Klebsiella* were associated with resistance to penicillins and cephalosporins. And for the aminoglycoside transferases WP_000018329.1 (aminoglycoside *O*-phosphotransferase APH(3′)-Ia), WP_032491824.1 (ANT(3″)-Ia family aminoglycoside nucleotidyltransferase AadA22), and WP_000557454.1 (aminoglycoside *N*-acetyltransferase AAC(3)-IId), the top hits in *K*. *pneumoniae* were associated with resistance to aminoglycosides. Moreover, all three chromosome-derived CDSs (WP_004176269.1, WP_076027158.1, and WP_004146118.1) matched to centroids found in >92% of the *K*. *pneumoniae* isolates, two of the five plasmid-derived CDSs (WP_032491824.1 and WP_000027057.1) matched to centroids with a frequency of >25%, while the remaining CDSs matched to centroids with a frequency of <12%.

## Discussion

To facilitate the studies on antibiotic resistance, we have built GEAR-base, a resource incorporating paired data on resistance phenotypes and genomic features for an extensive, longitudinal collection of clinical isolates from various bacterial species. This concerted effort is expected to reduce experimental bias and the present resource provides a portal for information retrieval as well as data analysis.

Species-level antibiotic resistance phenotypes can be inspected using the culture-based module in GEAR-base. Specifically, resistance rates and trends across multiple species and antibiotic drugs can be assessed on a large scale, which we believe is important for current and future antibiotic resistance research. Although some effect of potential sampling bias cannot be excluded, our findings on the increased resistance rates corroborate previously reported trends. In addition to this phenotypic information, genomic information is included in the pan-genome module. Such information can be used independent of the phenotypic information, *i.e.*, purely from a pan-genomic perspective, *e.g.*, for the study of inter- or intra-species gene conservation. The observed number of core centroids was consistent with the statistics reported by panX. However, GEAR-base pan-genomes are based on significantly higher sample number and are substantially larger in size, thus giving access to a comprehensive collection of the genome heterogeneity for human bacterial pathogens. In addition, GEAR-base links these two information layers through centroid–drug associations. These associations can subsequently be explored to study resistance mechanisms. Furthermore, individual researchers can compare genes or genomes of interest to the present resource, thereby providing an independent layer of support. This functionality was demonstrated using a recently published carbapenem-resistant *K. pneumoniae* isolate. While the taxonomic classifications of the genome and of a set of chromosome-derived antibiotic resistance genes are consistent with the expected taxonomy of the isolate, the plasmid-derived antibiotic resistance genes exhibit ambiguous taxonomic assignments, which is not unexpected for plasmid-borne genes. Moreover, the extensive collection of isolates included herein enables the study of the overall conservation degrees and the time-resolved frequencies of this exemplary antibiotic resistance gene set.

The analysis functionality in GEAR-base covers external genome databases (NCBI RefSeq as well as the NCTC 3000 genomes project from Public Health England and the Wellcome Trust Sanger Institute) in addition to the present collection of clinical isolate genomes. However, because the majority of external genomes are not linked to antibiotic resistance information and centroid–drug associations are considered a key component of the present resource, the pan-genome module is restricted to the present isolates. Additionally, the species-level pan-genome centroids in GEAR-base are available for download and provide a great opportunity for subsequent integration with external genomes for further study.

Emerging antibiotic resistance represents a multi-disciplinary and global challenge. We believe that GEAR-base will serve as a valuable resource enabling the detailed analysis of resistance-associated genomic features. GEAR-base includes a comprehensive selection of clinically highly relevant human microbial pathogens and will thus be of great use for the research and clinical communities.

## Materials and methods

### Bacterial isolates

The dataset of 11,087 isolates consisted of 1001 isolates from the *S*. *aureus* strain collection of Saarland University Medical Center and a collection of 10,086 Gram-negative bacterial clinical isolates that form part of the microbiology strain collection of Siemens Healthcare Diagnostics (West Sacramento, CA) [32]. DNA extraction using the Siemens VERSANT® sample preparation system [41] and whole-genome next-generation sequencing were performed for all isolates as described in Galata et al. [32] (2 × 100 bp paired-end on Illumina Hiseq2000/2500 sequencers).

### Methicillin susceptibility of *S*. *aureus* isolates

For 993 isolates from the *S*. *aureus* strain collection, detection of methicillin-resistant or methicillin-susceptible *Staphylococcus aureus* (MRSA/MSSA) isolates was performed. The specimen were plated on CHROMagar MRSA detection biplates (Mast, Reinfeld, Germany). All MRSA-positive culture isolates were further confirmed using a penicillin-binding protein 2a latex agglutination test (Alere, Köln, Germany).

### Susceptibility testing and resistance profiles of Gram-negative isolates

For 9998 isolates from the Gram-negative isolate collection, AST was performed. Frozen reference AST panels were prepared following Clinical Laboratory Standards Institute (CLSI) recommendations [42]. The antimicrobial agents included in the panels are provided in Table S2. Prior to use with clinical isolates, AST panels were tested and considered acceptable for testing with clinical isolates when the QC results met QC ranges described by CLSI [42].

Isolates were cultured on trypticase soy agar with 5% sheep blood (Bethesda Biological Laboratories, Cockeysville, MD) and incubated in ambient air at 35 ± 1 °C for 18–24 h. Isolated colony panels were inoculated according to the CLSI recommendations (CLSI additional reference) and incubated in ambient air at 35 ± 1 °C for 16–20 h. Panel results were read visually, and MICs were determined.

#### MIC value processing

The bacterial culture may not grow for the lowest drug concentration tested (expressed as ≤*x*) or show no significant growth decrease for the highest concentration tested (expressed as >*x*), where *x* represents the drug concentration tested. To allow consistent processing, these MIC values were transformed as follows: in the former case, the MIC value was set to be *x*/2 (*e.g.*, “≤0.25” was set to “0.125”), and in the latter case, the MIC value was set to be *x* * 2 (*e.g.*, “>64” was set to “128”). Additionally, we considered only the MIC value of the first agent in case of drug combinations (*e.g.*, “32/16” was set to “32”).

#### Drug information

The 21 drugs used in this study were grouped into 8 drug classes based on their category in the EUCAST guidelines [43]. Among them, 7 drugs belong to cephalosporins (cefazolin and cephalotin – 1st generation; cefuroxime – 2nd generation; cefotaxime, ceftazidime, and ceftriaxone – 3rd generation; and cefepime – 4th generation), 4 to penicillins, 3 to carbapenems, 2 to fluoroquinolones, 2 to aminoglycosides, and 1 to tetracycline. In addition, 1 drug is a monobactam and the remaining 1 drug falls into the category “miscellaneous” (Table S2).

#### Resistance classification

EUCAST guidelines [43] (v. 4.0) were used for MIC value classification. Isolates were classified as resistant, intermediate, or susceptible. An isolate was considered to be resistant if the corresponding MIC value was greater than the resistance breakpoint. If the MIC value was below or equal to the susceptibility breakpoint, the isolate was considered to be susceptible. If the MIC value was between the two breakpoints, the isolate was considered as “intermediate”. If no breakpoint was available for a specific drug and bacterial group, no classification was performed.

### Genome-based taxonomic classification

Kraken [44] (v. 0.10.4-beta) was used with the default database containing finished genomes from the NCBI RefSeq database (accessed on January 13, 2015) and a k-mer length of 31. Report files were created from the raw output using “kraken-report” and processed to retrieve the information, including (1) the first best species hit relative to the percentage of mapped sequences; (2) the number of sequences mapped to best hit; (3) the number of sequences classified at species level; (4) the number of unclassified sequences; and (5) the total number of reported sequences. In addition, sensitivity values, precision values, and percentages of unassigned sequences were calculated. Sensitivity was defined as the ratio of reads assigned to the best hit over the total number of reported reads. Precision was defined as the ratio of reads assigned to the best hit over reads classified at species level. For each sample, the taxonomic lineage from the species to the class level was retrieved using the R package “taxize” [45] and the NCBI [46] taxonomy database (accessed on February 8, 2016). An overview of the taxonomic composition of the dataset was created using Krona [47].

### Read processing and assembly pipeline

The raw sequencing reads were trimmed using Trimmomatic [48] (v. 0.35, command line parameters: PE ILLUMINACLIP:NexteraPE-PE.fa:1:50:30 LEADING:3 TRAILING:3 SLIDINGWINDOW:4:15 MINLEN:36). Trimmed paired-end reads were assembled *de novo* into scaffolds (from now on called contigs for simplicity) using SPAdes [49] (v. 3.6.2, parameters: -k 21,33,55 --careful) and annotated by Prokka [50] (v. 1.11, parameters: --gram neg --mincontiglength 200). Assembly quality was assessed using QUAST [51] (v. 3.2, parameters: --contig-thresholds 0,100,200,500,1000 --min-contig 200).

#### Mean assembly coverage

Trimmed reads were mapped to the contigs (minimal length of 200 bp) using BWA [52] (v. 0.7.12) and SAMtools [53] (v. 1.2; command line: bwa mem –M –t <cores> <contigs> <forward reads> <reverse reads> | samtools view @ <cores> -bt <contigs> - | samtools sort -@ <cores> - <bam>). Then coverage histogram was computed using BEDtools [54] (v. 2.25; parameters: bedtools genomecov –ibam <bam>-g <contigs> > <hist>). Finally mean coverage was computed over all contigs.

#### Essential genes

Essential genes as defined by Dupont et al. [33] were downloaded (https://github.com/MadsAlbertsen/multi-metagenome/raw/master/R.data.generation/essential.hmm, accessed on March 7, 2017) and searched in the present assemblies (protein FASTA files of translated CDS; *.faa) using hmmsearch from the HMMER software package (http://hmmer.org/, v. 3.1b2, parameters: --cut-tc). Only hits with at least one domain satisfying the reporting thresholds (column “rep” in table output files) were considered. Best hits for each isolate and essential gene were determined with respect to the E-value of reported full sequences. Finally, each considered hit was assigned to a centroid, *i.e.*, the centroid covering the gene from the corresponding hit.

#### Resistance factors

The Resfams core database [34] of HMMs (v1.2) was used to identify known resistance factors in the present assemblies (*.faa, FASTA file of protein annotations) using hmmsearch from the HMMER software package (http://hmmer.org/, v. 3.1b2, parameters: hmmsearch --cut_ga --tblout output.tblout Resfams.hmm input.faa > output.hmmout).

MLST profiles were determined using the BLASTn search-based tool mlst (https://github.com/tseemann/mlst, accessed on August 8, 2016, v. 2.9, parameters: --minid 99 --mincov 75 --minscore 99) on assembled contigs (minimal length of 200 bp).

### Sample filtering

First, the bacterial isolate samples were filtered on the basis of their taxonomic assignment and assembly quality. For the taxonomic assignments, the minimal sensitivity was set to 50% (0% for *Shigella*), the minimal precision to 75% (60% for *Shigella*), and the minimal percentage of unclassified reads to 30%. The cutoff values were “relaxed” for *Shigella* because of the well-known problem of high genetic similarity between the *Shigella* species and *E*. *coli* [55], making it difficult to differentiate between these organisms at the nucleotide level, which affects the taxonomic sensitivity. For the *de novo* assemblies, we used the criteria defined by RefSeq [37]: number of contigs ≤1000, N50 ≥5000, and L50 ≤200. Isolates that passed both filtering steps were grouped by their species taxon, and only species containing at least 50 isolates were further considered. As a result, the following 18 species (referred to as “main species” in the manuscript) passed the filtering step. These include *A. baumannii*, *B. cenocepacia*, *Citrobacter koseri*, *E. aerogenes*, *E. cloacae*, *E. coli*, *K. oxytoca*, *K. pneumoniae*, *M. morganii*, *P. mirabilis*, *P. aeruginosa*, *S. enterica*, *S. marcescens*, *Shigella boydii*, *S. flexneri*, *S. sonnei*, *S. aureus*, and *S. maltophilia*. Additionally, samples containing more than 10 essential genes in multiple copies were examined further by running Kraken (*k* = 31) on the nucleotide sequences of the annotated genomic features (*.ffn). Report files were created from filtered assignments (kraken-filter, threshold 0.05) and inspected manually in order to determine whether a large percentage of sequences was assigned to unexpected species. In total, 8729 isolates remained assigned to the 18 main species mentioned above.

### Pan-genome construction

Roary [17] (v. 3.5.7, parameters: -e -n -i 90 -cd 90 -a -g 70,000 -r -s -t 11) was used to construct the species-level pan-genomes.

#### Centroid HMMs

The protein sequences were extracted from the FASTA files of translated CDS (*.faa) created by Prokka [50]. For non-CDS sequences, protein sequences were created by translating the corresponding nucleotide sequences from the nucleotide FASTA files (*.ffn) using BioPython (parameters: table = 11, stop_symbol=“*”, to_stop = False, cds = False). Multiple sequence alignments were created using MUSCLE [56] (v. 3.8.31, parameters: -maxiters 1 -diags -sv -distance1 kbit20_3). HMM profiles were calculated using hmmbuild from the HMMER software package (http://hmmer.org/, v. 3.1b2).

### Database

The GEAR-base was implemented using the Python web framework Django (v. 1.9.5) and MySQL (v. 15.11) as the database management system. HMM search in Resfams core database and centroid HMM profiles is implemented using package/library HMMER (http://hmmer.org/, v. 3.1b1). Moreover, sketches of centroid nucleotide sequences were computed using Sourmash [35] (v. 2.0.0.a1, sketching parameters: sourmash compute --dna --singleton --scaled 10 --seed 42 --ksizes 21, indexing parameters: sourmash index --dna --ksize 21). Mash/MinHash [38] (v. 1.1.1, default parameters) was used to create sketches of GEAR-base isolates, finished bacterial genomes from the NCBI RefSeq database, and assembled bacterial genomes from the NCTC 3000 database of Public Health England and the Wellcome Trust Sanger Institute. The genomes from the NCBI RefSeq database included 7118 genomes and were downloaded on June 17, 2017 using the NCBI genome downloading scripts of Kai Blin (https://github.com/kblin/ncbi-genome-download, accessed on October 18, 2017, v. 0.2.2) with the setting “ncbi-genome-download --section refseq --assembly-level complete --human-readable --parallel 10 --retries 3 --verbose bacteria” with “--format fasta” and “--format cds-fasta”). The bacterial genomes from the NCTC 3000 database were downloaded on July 10, 2017 and included 1052 genomes.

### Resistance profile analysis of cultured isolates from the Gram-negative collection

#### Drug correlations

Considering only species with ≥50 isolates, pairwise drug correlations were computed using the MIC value profiles (Spearman’s correlation coefficient, all isolates and for each species taxon separately). Drugs with a single MIC value across all considered isolates were removed prior to correlation computation. To visualize possible drug–drug associations, hierarchical clustering using Euclidean distance and average linkage was applied.

#### Association between isolate collection year and resistance profiles

Two-sided WMW-test (R package exactRankTests, v. 0.8-29) was applied to the isolates with assigned collection year available and belonging to a species taxon with ≥50 isolates (in total 8768 isolates from 18 taxa). The isolates were divided into resistant and non-resistant (susceptible and intermediate) groups. No test was performed if either group included <10 isolates or all isolates in a group were collected in the same year. All *P* values were adjusted using FDR.

### Phylogenetic analysis

Essential genes, found in ≥99% of the isolates that were used to construct the pan-genomes, were identified. Protein sequences for the corresponding best hits were extracted for each essential gene and isolate. Multiple sequence alignments were computed using MUSCLE [56] (v. 3.8.31, parameters: -maxiters 1 -diags -sv -distance1 kbit20_3) for each essential gene separately and concatenated into one alignment. If an isolate did not have any matches, an empty alignment sequence (*i.e.*, containing only gap characters) was added. RAxML [57] (v. 8.2.9, raxmlHPC-PTHREADS) was used to construct a phylogenetic tree from the aggregated alignment. After removing sequence duplicates (2297 in total) and alignment columns containing only undetermined values, *i*.*e*. ambiguous characters, (147 in total), the tree was built using the CAT model (parameters: -p 12,345 -m PROTCATAUTO -F -T 30).

### Pan-genome analysis

#### Centroid rate estimation

The centroid presence–absence tables created by Roary were used to estimate the median number of total, new, unique, and core centroids in species-level pan-genomes relative to the number of isolates used (rarefaction). For each pan-genome, the columns (isolates) of the table were permuted 100 times. Starting from the first isolate, centroid counts were calculated in a cumulative manner for each permutation. The centroid categories were defined as follows: total centroids comprise centroids found in at least one of the included genomes; new centroids refer to the centroids found only in the last included genome; unique centroids are centroids found only in one of the included genomes; and core centroids are centroids found in ≥90%, ≥95%, and ≥99% of all included genomes to cover different levels of conservation. The median centroid counts were computed over all permutations. The curve of the total number of centroids was fitted using nonlinear least-squares estimates (R method “nls”) of the power law function $n=a\cdot N^{\gamma }$ (where n is the total number of centroids, N is the number of included genomes, and a and γ are constants) to the median counts.

#### Two-dimensional embedding of pan-genome centroids

BusyBee Web [58] was used to represent the pan-genome centroids in two dimensions (2D). In brief, pentanucleotide frequencies were computed and transformed into 2D using Barnes–Hut stochastic neighbor embedding [59]. Due to the use of centroids rather than contigs or long reads, the border point threshold and cluster point threshold were set to 500. Individual pan-genomes were mixed *in silico*, centroids with a frequency ≥90% were used as input to BusyBee Web, and the 2D coordinates were downloaded. Here, in addition to the sample frequency overlay, centroids were colored according to the respective species of the source pan-genome of the centroid.

### Resistance association analysis

#### Association between resistance profiles and centroid presence

All isolates that were used to construct the pan-genomes and had resistance profiles available were considered. Binary centroid presence/absence matrices were used as features. A species–drug combination was not analyzed if >90% of the isolates were resistant or non-resistant. The predictors were first filtered to remove (nearly) constant and correlated features and features with many missing values. All predictors with >95% missing values or with >95% of the entries having the same value (missing values ignored) were removed. Correlated features were removed by computing pairwise feature correlations (fastCor from R-package HiClimR, v. 1.2.3), clustering them using hierarchical clustering (distance = 1 – cor^2, average linkage), cutting the resulting tree at height 0.0975 (1–0.95^2^), and keeping only medoids (minimal average distance to other cluster members) within each obtained cluster. All features were scored using EIGENSTRAT [60] (v. 6.0.1) to correct for possible population structures. First, principal component analysis (PCA) was run to compute the top 50 principal components using only retained features. Then, the number of components (k) used for the subsequent computation was chosen such that the estimated genomic inflation factor (lambda) was <1.1 for the smallest possible k. If none of the computed lambda values was <1.1, then k with the smallest lambda value was chosen. The value of k was successively increased from k = 1 to k = 50 by an increment of 2. With the chosen value of k, test statistics were generated for all features and P values were computed using the Chi-squared distribution with one degree of freedom. Finally, FDR adjustment was applied*.*

#### Number of Resfams covered by the significant resistance association results

For each centroid with a significant resistance association result (adjusted *P* < 1E−5), all hits from the centroid cluster members to the Resfams core database were retrieved. Subsequently, for each Resfam, the number of unique centroids including ≥1 cluster member with a hit to the corresponding Resfam was counted.

### Application example

The assembly of the complete *K*. *pneumoniae* genome published by Kao et al. [39] (NCBI assembly No. ASM195283v1, RefSeq assembly accession No. GCF_001952835.1) was included in the collection of the finished bacterial genomes downloaded from the NCBI RefSeq database as described above. The genomic FASTA file containing the chromosome and plasmid sequences was uploaded to the GEAR-base web-server for genome analysis using default parameters (https://gear-base.com/gear/pangenome/genomesearch/job=b568c458-f68a-4aa1-b78b-dad72dddfd5a/). The FASTA file containing the nucleotide sequences of all CDSs was uploaded for gene-based analysis with only Resfams search and Sourmash search in centroids enabled and using default parameters (https://gear-base.com/gear/pangenome/genesearch/job=0e42e149-a70d-4796-b40a-7f7168dc5077/). The nucleotide sequences of eight resistance genes reported previously [39], including WP_004176269.1, WP_076027158.1, WP_004146118.1, WP_000018329.1, WP_032491824.1, WP_000557454.1, WP_000976514.1, and WP_000027057.1, were saved in a separate FASTA file, which was uploaded for gene-based analysis with all options enabled and default parameters (https://gear-base.com/gear/pangenome/genesearch/job=d8792c0e-bbe7-4936-a7b7-c2846b727afe/).

## Availability

GEAR-base is freely available for academic research use after the user has registered and accepted the terms of use available at https://gear-base.com. Because of the sheer size and further legal and ethical constrains, we cannot make all data fully accessible for batch download. If users are interested in getting access to the raw sequencing data, a special request in this respect is required. For this, we provide a respective request details on the GEAR-base homepage. The sequences of pan-genome centroids can be downloaded directly from the GEAR-base homepage. Custom scripts used for processing, analyzing and plotting the data can be found at https://github.com/VGalata/gear_base_scripts/.

## Authors’ contributions

VG performed the computational analysis, implemented the database, and drafted the manuscript together with CCL. CCL and CB also contributed to the data analysis. GH-S and AF performed the next-generation sequencing of the isolates. MH and LvM provided the *S*. *aureus* isolate collection. AEP, SS, CS, and AP provided the Gram-negative isolate collection. EM, RM, and AK reviewed the manuscript and provided comments. All authors read and approved the final manuscript.

## Competing interests

CS and AEP were employees of Siemens Healthcare during the period of the study. SS is an employee of Siemens Healthcare. AEP and AP are Managing Directors of Ares Genetics GmbH, a wholly owned subsidiary of Curetis GmbH. Ares Genetics GmbH is the sole owner of any and all rights to the data presented in the manuscript and in the web resource at https://gear-base.com. Those who are interested in commercial applications or collaboration are invited to contact Ares Genetics at contact@ares-genetics.com.

## Supplementary material

Supplementary Figure S2 **Drug correlations** Heatmaps of the drug correlation matrices based on MIC value profiles. The first plot corresponds to correlation matrix computed using all isolates of selected species taxa; the remaining plots were generated for each species separately. Only species with at least 50 isolates were included. The cells in the heatmap are colored from blue to red with blue for lower correlation values and red for higher correlation values ranging from −1 to 1. The drugs were ordered using hierarchical clustering (Euclidean distance and average linkage) and colored with respect to their class.

Supplementary Figure S9 **MLST analysis** Pie charts showing the proportion of found MLSTs. The top 5 known STs are shown, known STs not included in the top 5 were grouped into ”other”, ”new” after the name of the scheme refers to new STs, and ”unknown” refers to cases where no matching scheme was found. MLST, multi-locus sequence typing.
