# Supplementary material for: Integrating Culture-based Antibiotic Resistance Profiles with Whole-genome Sequencing Data for 11,087 Clinical Isolates
Source: Genomics Proteomics Bioinformatics. 2019 May 14;17(2):169–82. doi: 10.1016/j.gpb.2018.11.002 (PMC6624217; doi:10.1016/j.gpb.2018.11.002)
Supplement: Supplementary Figure S2 — Drug correlations Heatmaps of the drug correlation matrices based on MIC value profiles. The first plot corresponds to correlation matrix computed using all isolates of selected species taxa; the remaining plots were generated for each species separately. Only species with at least 50 isolates were included. The cells in the heatmap are colored from blue to red with blue for lower correlation values and red for higher correlation values ranging from −1 to 1. The drugs were ordered using hierarchical clustering (Euclidean distance and average linkage) and colored with respect to their class. [file mmc1.pdf]

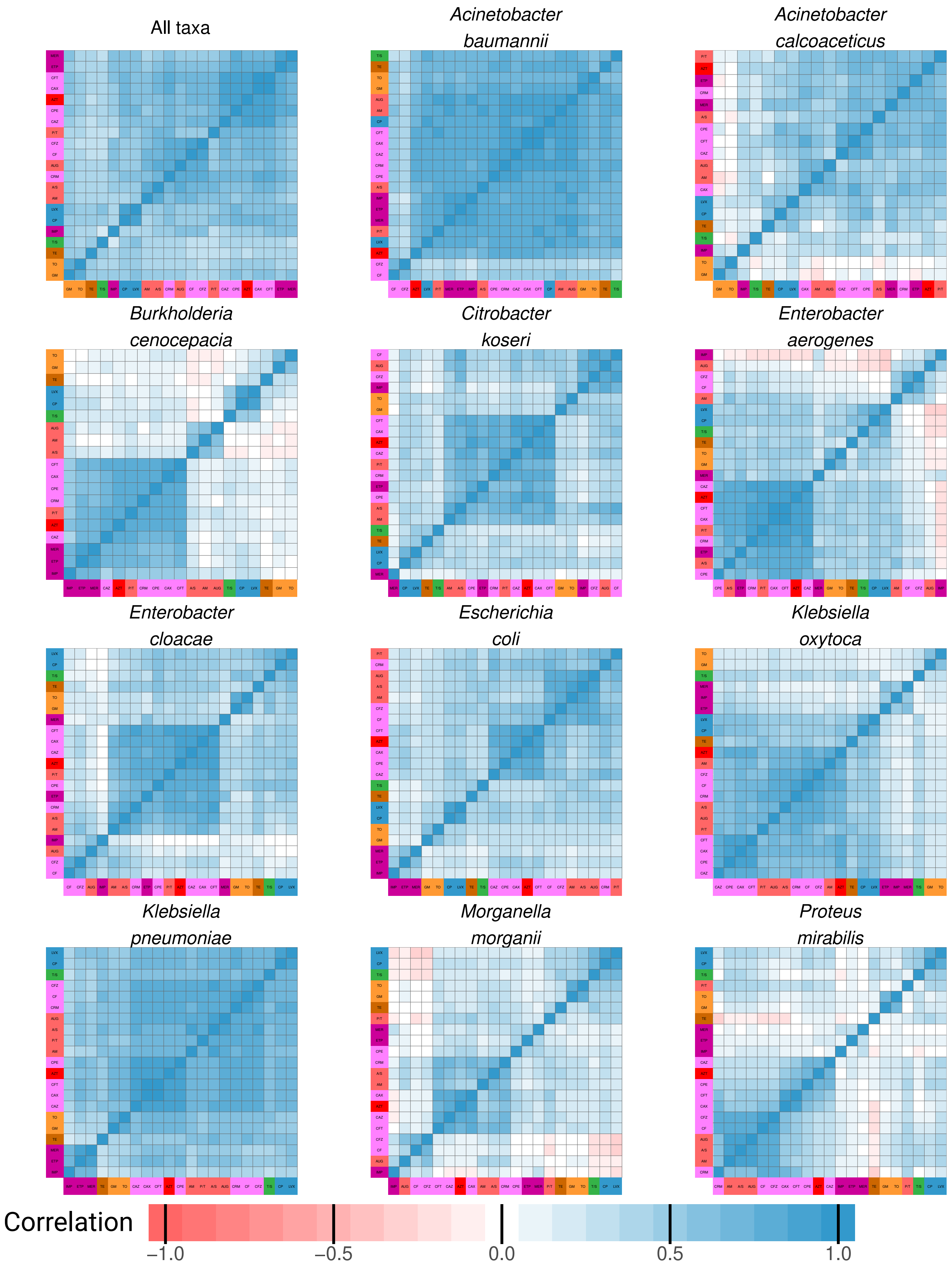
All taxa
Acinetobacter baumannii
Acinetobacter calcoaceticus
Burkholderia cenocepacia
Citrobacter koseri
Enterobacter aerogenes
Enterobacter cloacae
Escherichia coli
Klebsiella oxytoca
Klebsiella pneumoniae
Morganella morganii
Proteus mirabilis
Correlation
−1.0
−0.5
0.0
0.5
1.0

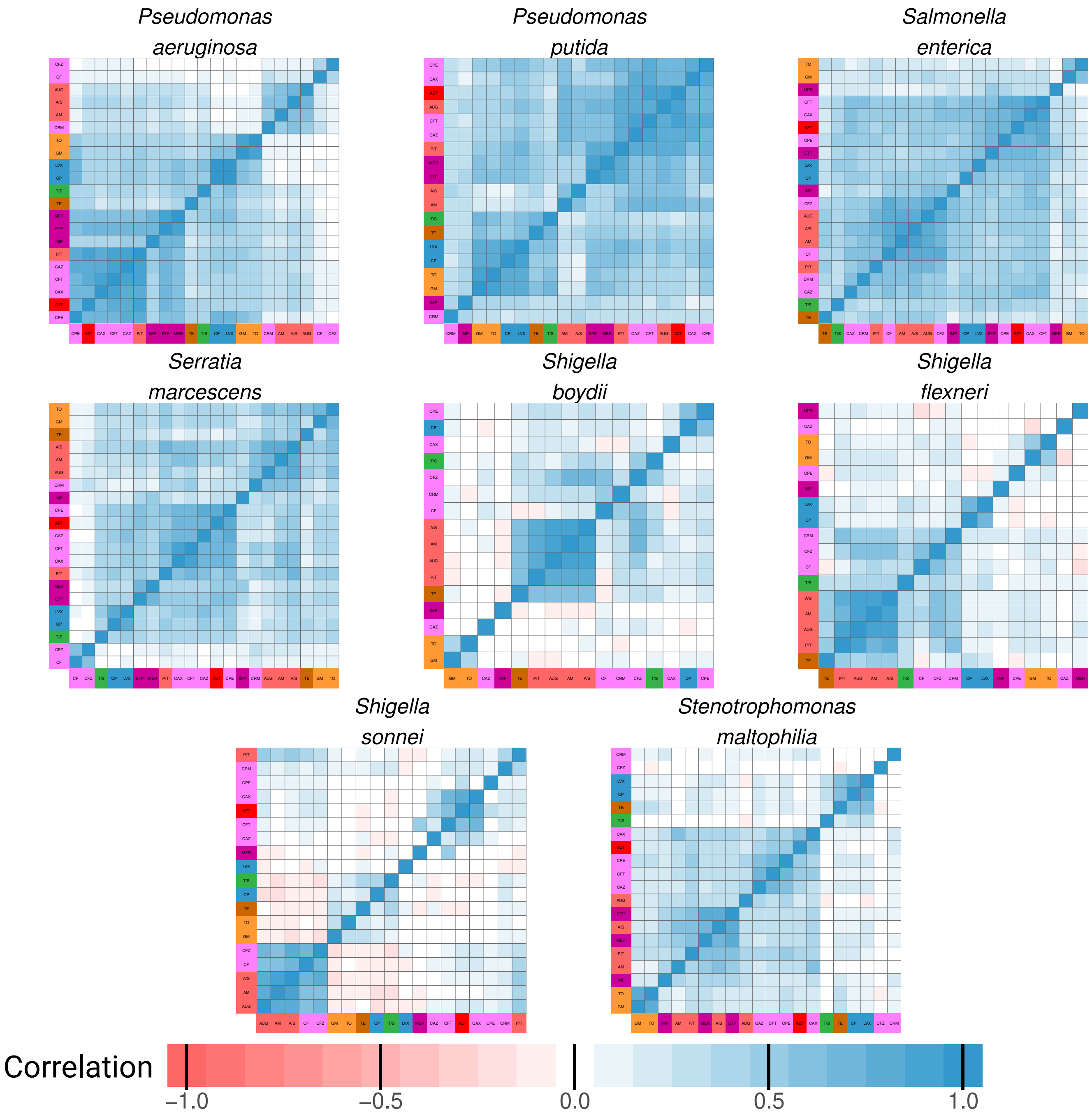

Pseudomonas aeruginosa
Pseudomonas putida
Salmonella enterica
Serratia marcescens
Shigella boydii
Shigella flexneri
Shigella sonnei
Stenotrophomonas maltophilia
Correlation
−1.0
−0.5
0.0
0.5
1.0
