# Supplementary material for: Integrating Culture-based Antibiotic Resistance Profiles with Whole-genome Sequencing Data for 11,087 Clinical Isolates
Source: Genomics Proteomics Bioinformatics. 2019 May 14;17(2):169–82. doi: 10.1016/j.gpb.2018.11.002 (PMC6624217; doi:10.1016/j.gpb.2018.11.002)

Acinetobacter baumannii

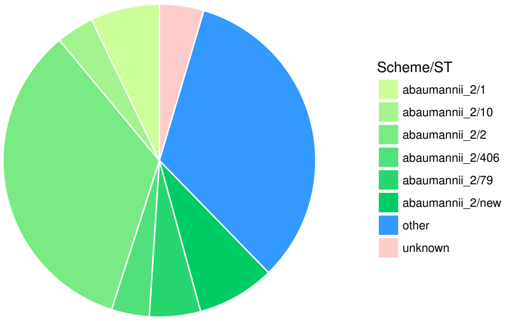

Burkholderia cenocepacia

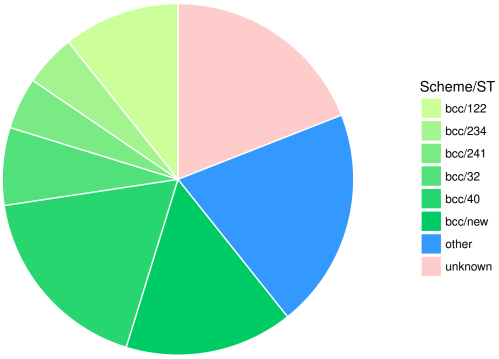

Citrobacter koseri

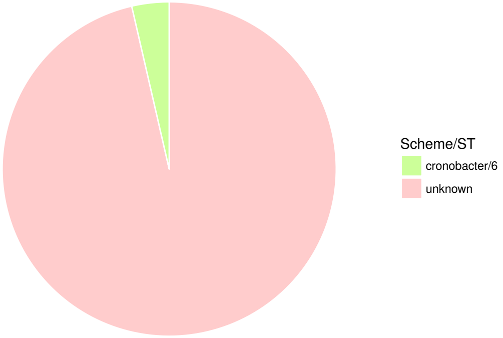

Enterobacter aerogenes

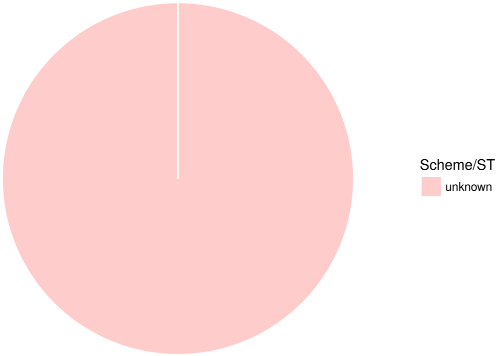

Enterobacter cloacae

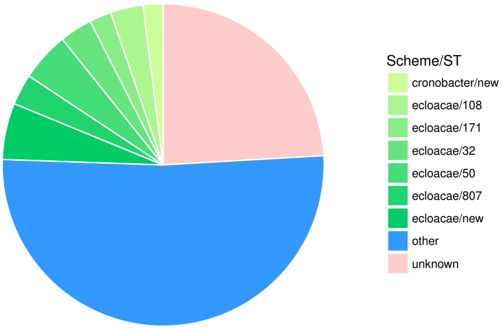

Escherichia coli

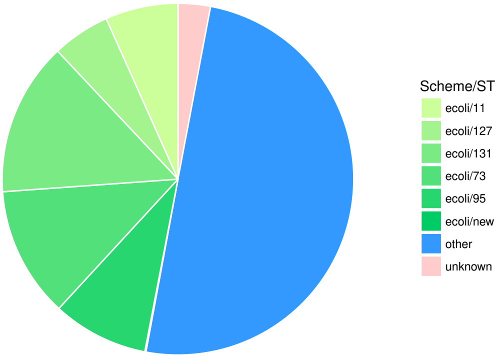

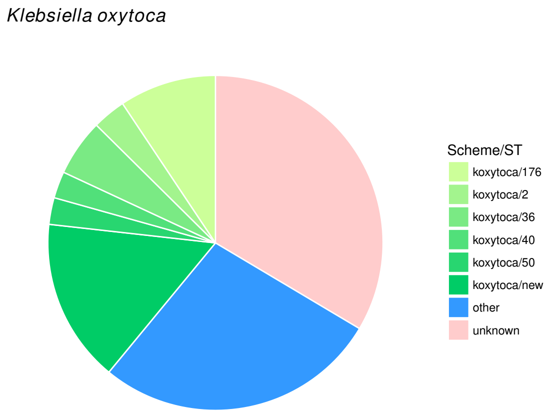
Klebsiella oxytoca
Scheme/ST
koxytoca/176
koxytoca/2
koxytoca/36
koxytoca/40
koxytoca/50
koxytoca/new
other
unknown

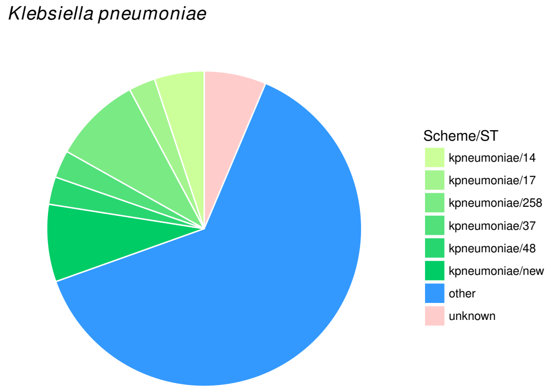
Klebsiella pneumoniae
Scheme/ST
kpneumoniae/14
kpneumoniae/17
kpneumoniae/258
kpneumoniae/37
kpneumoniae/48
kpneumoniae/new
other
unknown

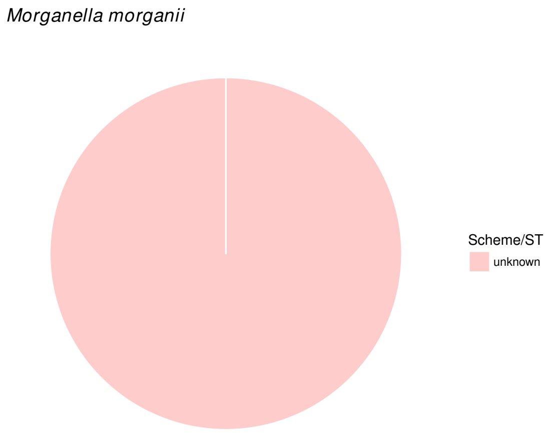
Morganella morganii
Scheme/ST
unknown

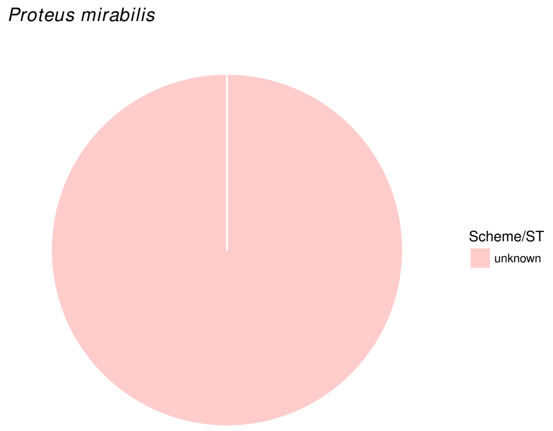
Proteus mirabilis
Scheme/ST
unknown

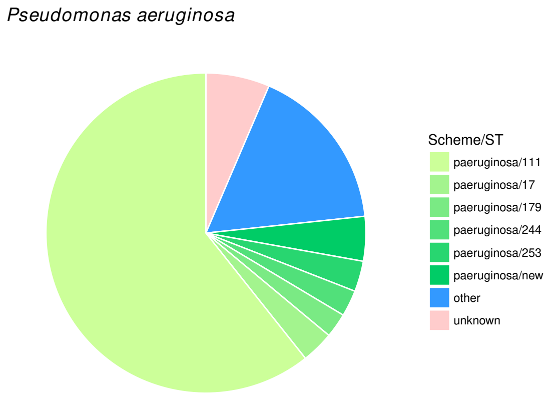
Pseudomonas aeruginosa
Scheme/ST
paeruginosa/111
paeruginosa/17
paeruginosa/179
paeruginosa/244
paeruginosa/253
paeruginosa/new
other
unknown

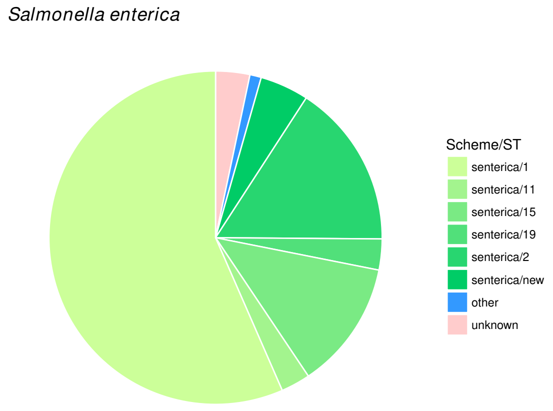
Salmonella enterica
Scheme/ST
senterica/1
senterica/11
senterica/15
senterica/19
senterica/2
senterica/new
other
unknown

Serratia marcescens

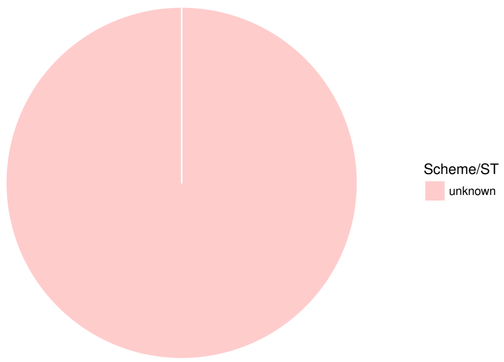

Shigella boydii

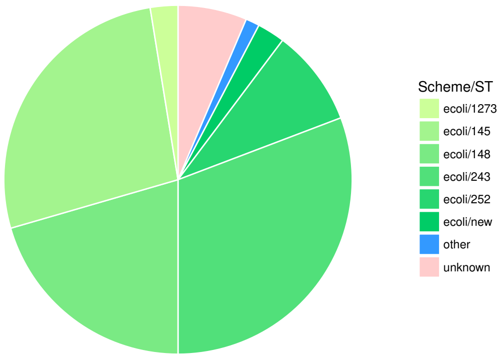

Shigella flexneri

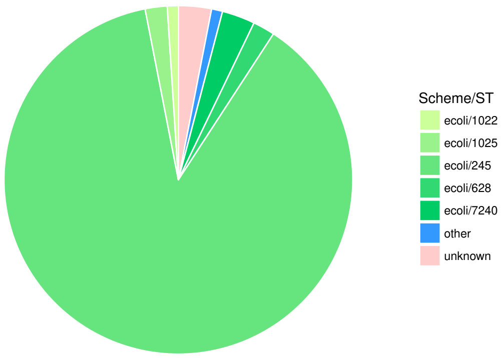

Shigella sonnei

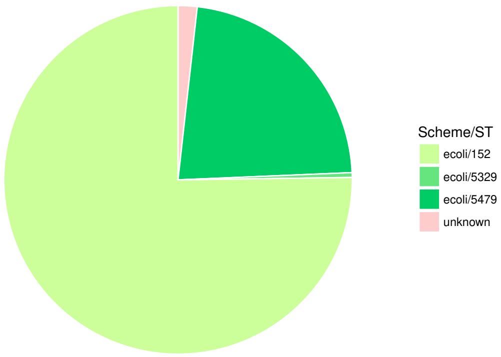

Staphylococcus aureus

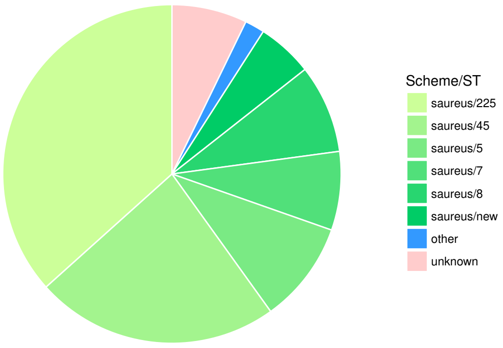

Stenotrophomonas maltophilia

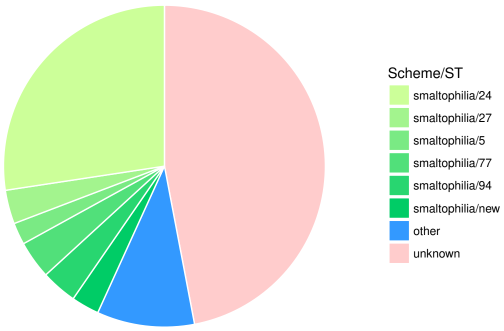

Supplement: Supplementary Figure S9 — MLST analysis Pie charts showing the proportion of found MLSTs. The top 5 known STs are shown, known STs not included in the top 5 were grouped into ”other”, ”new” after the name of the scheme refers to new STs, and ”unknown” refers to cases where no matching scheme was found. MLST, multi-locus sequence typing. [file mmc2.pdf]
